# Advanced Glycation End Products Mediate Epigenetic Alteration of H3K27me3 in Renal Proximal Tubular Cells: Potential Role in Metabolic Memory

**DOI:** 10.3390/cells14211729

**Published:** 2025-11-04

**Authors:** Lore Ludewig, Tzvetanka Bondeva, Marita Liebisch, Jonas Ihle, Ivonne Loeffler, Gunter Wolf

**Affiliations:** Department of Internal Medicine III, University Hospital Jena, Am Klinikum 1, 07747 Jena, Germany; lore.ludewig@uni-jena.de (L.L.); marita.liebisch@med.uni-jena.de (M.L.); jonas.ihle@gmx.de (J.I.); ivonne.loeffler@med.uni-jena.de (I.L.); gunter.wolf@med.uni-jena.de (G.W.)

**Keywords:** AGEs, advanced glycated end products, metabolic memory, EZH2, enhancer of zeste homolog 2, H3K27me3, histone 3 K27 tri-methylation, *db/db* mice, DZNep, 3-deazaneplanocin A

## Abstract

**Highlights:**

**What are the main findings?**

- AGE-BSA reduces EZH2 expression and the repressive histone mark H3K27me3 in proximal tubular TKPTS cells, leading to increased expression of genes involved in renal injury, including *Ctgf*, *Snai1*, and *p27^Kip1^*.
- Pharmacological inhibition of EZH2 with DZNep mimics and enhances these effects, and in vivo studies show reduced H3K27me3 in proximal tubules of diabetic mice, with sex-specific differences in EZH2 expression.

**What is the implication of the main finding?**

- The suppression of EZH2 by AGEs represents an epigenetic mechanism contributing to proximal tubular cell damage and the progression of diabetic nephropathy.
- These results provide insight into metabolic memory, highlighting that prior hyperglycemia can induce persistent epigenetic changes and sex-specific vulnerabilities in the kidney, even under current normoglycemia.

**Abstract:**

The accumulation of advanced glycation end products (AGEs) is a hallmark of prolonged high glucose levels in diabetes mellitus. We have previously reported that hypoxia and AGEs cause epigenetic modification of the repressive mark H3K27me3 in podocytes by downregulation of enhancer of zeste homolog 2 (EZH2) and nuclear inhibitor of protein phosphatase 1 (NIPP1). However, their impact on proximal tubular cells remains unclear. The aim of this study was to investigate the role of AGEs and diabetes on the epigenetic modifications of EZH2 and H3K27me3 in proximal tubular cells and in diabetic (*db/db*) mice. Our results show that AGEs reduced EZH2 expression in TKPTS cells, thereby decreasing the tri-methylation of H3K27. qRT-PCR analysis revealed upregulation of genes known to contribute to diabetic nephropathy and kidney injury as *Ctgf*, *Snai1*, and *p27^Kip1^*. Consistently, immunofluorescent staining of renal sections from *db/db* mice confirmed the reduction in H3K27me3 levels in proximal tubules compared to non-diabetic controls. In summary, we show that AGEs induce epigenetic changes in proximal tubular cells by suppressing EZH2, thereby facilitating the transcription of genes involved in progression of diabetic nephropathy. These results provide new insights into metabolic memory, a process in which prior poor glucose control triggers ongoing renal damage despite current normoglycemia.

## 1. Introduction

Advanced glycated end products (AGEs) are posttranslational modification generated via non-enzymatic glycation of lipids, proteins, or nucleic acids through reducing sugars (Maillard reaction). Prolonged, uncontrolled hyperglycemia enhances AGE formation and contributes to development of diabetic nephropathy [1,2], promotes the process of epithelial–mesenchymal transition via TGF-β production [3] and many diabetes-related complications [4,5,6,7]. According to Chilelli et al. [8] AGEs play a more important role in microvascular complication in diabetes than hyperglycemia [8]. Even after the abnormal glucose is under control, or other metabolic abnormal environmental conditions return to basal levels, the cellular stress persists as a “metabolic memory”, reflecting early metabolic insult [9]. In this regard, AGEs may represent such persistent molecular fingerprints, as glycated-proteins, nucleic acid, or lipids remain far longer in the cells than transient hyperglycemia. Therefore, AGEs may contribute to the metabolic or glycemic memory phenomenon. Aberrant epigenetic modifications could serve as additional cellular fingerprints of transient metabolic disorders.

The epigenetic regulation including DNA methylation and histone modifications such as methylation, acetylation, or deacetylation were considered as a potent factor that can alter gene expression independently of DNA sequence [10]. An important role in the regulation of the gene expression through epigenetic modification was reported for the repressive histone mark histone 3 K27 (H3K27) tri-methylation (H3K27me3) that functions as a transcriptional repressor of genes involved in development and differentiation [11,12,13,14]. The epigenetic alteration of the tri-methylation of the repressive mark H3K27me3 unlocked the expression of genes that contribute to pathological changes and renal damage [15]. The enhancer of zeste homolog (EZH2) is the catalytic subunit of the polycomb-repressive complex 2 (PRC2), which possesses a methyltransferase activity and mediates specifically mono-, di-, and tri-methylation of histone 3 K27 [11,16].

The role of EZH2 in regulating the epigenetic changes of H3K27me3 has been previously studied in various renal cell types and experimental conditions. Previously, it was shown that, high glucose inhibits Wilm’s tumor1 (WT1) and activates EZH2/β-catenin signaling pathway leading to podocytes injury in diabetic nephropathy [10], whereas Siddiqi et al. [17] have shown that EZH2 protects podocytes from oxidative stress and renal injury in diabetes [17]. We recently reported that AGEs reduce EZH2 expression in podocytes, lowering the repressive mark H3K27me3 and inducing the expression of *Snai1*, *p27^Kip1^*, and *Tgf-β*, thereby promoting podocytes damage and diabetic nephropathy [18]. Consistently, the expression of EZH2 and tri-methylation of H3K27 were decreased in kidney glomeruli of *db/db* mice compared with non-diabetic [18].

Furthermore, in renal epithelial cells and in the elder murine kidney, downregulation of the EZH2 promotes cellular senescence [19]. It was reported that 3-deazaneplanocin A (DZNep), an inhibitor of EZH2, protects proximal tubular epithelial cells (mTECs) from cisplatin-induced apoptosis in a mechanism independent of EZH2 or H3K27me3 [20]. On the other hand, in NRK-52E renal rat tubular epithelial cells, EZH2 inhibition by DZNep enhanced apoptosis by increasing mTOR interacting protein DEPTOR expression via H3K27me3-dependent mTORC1/2 inhibition [21].

Recently, an upregulation of miR-124 in serum samples from diabetic patients was detected [22]. Furthermore, elevated miR-124 levels inhibit insulin secretion and promote apoptosis in high-glucose stimulated transformed pancreatic beta cell line Min6 cells through suppression of EZH2 expression [23]. Consistent with this finding, the authors detected reduced EZH2 protein levels in pancreatic tissues of *db/db* mice, thus suggesting a crucial role of EZH2 and miR-124 in diabetic disease [23].

Despite this insight, EZH2 precise function and its regulation of the repressive mark H3K27me3 in both healthy and diabetic kidney remains unclear.

Therefore, the aim of our present study was to examine (I) whether AGEs mediate an epigenetic alteration on the repressive histone 3 mark H3K27me3 in proximal tubular cells and (II) are EZH2 expression and H3K27me3 levels affected from sex-difference in renal tubules of non-diabetic and *db/db* mice, a well-established type 2 diabetes model [24].

## 2. Materials and Methods

### 2.1. Cell Culture of TKPTS Cell Line

TKPTS cells (purchased from American Type Culture Collection (ATCC), Manassas, VA, USA) are a cell line generated from murine proximal tubular cells, originated from a male mouse [25]. The cells were cultivated according to the manufacturer’s instructions in Dulbecco’s Modified Eagle Medium containing 1g/L D-glucose (DMEM, Invitrogen, Darmstadt, Germany) supplemented with 10% heat-inactivated fetal calf serum (FCS; PAN Biotech GmbH, Aidenbach, Germany), 1% penicillin-streptomycin solution (Gibco™, Thermo Fisher Scientific, Darmstadt, Germany). The culture medium was supplemented with 6.3 µg/mL insulin solution (Sigma-Aldrich Chemie GmbH, Taufkirchen, Germany) according to manufacturer’s instructions. The cells were cultured at 37 °C with 5% CO_2_ supply. For experiments, TKPTS cells from passages 8 to 15 were used.

### 2.2. AGE-BSA Preparation and Treatment of TKPTS Cells

Glycated-BSA (AGE-BSA) and control, non-glycated-BSA (Co-BSA) were produced with fatty acid-poor, endotoxin-free BSA (Calbiochem, La Jolla, CA, USA), as previously reported [26]. All samples were dialyzed against PBS to remove the free glucose and lyophilized. Co-BSA and AGE-BSA were prepared just before treatments of the cells in DMEM containing 0.1% FCS, 1% penicillin–streptomycin and 6.3 µg/mL insulin solution. For analyses 50% confluent TKPTS cells were serum-deprived overnight, followed by treatment with 5 mg/mL Co-BSA and AGE-BSA, respectively, for 24 h. After that, the cells were washed with sterile PBS and used for subsequent analyses.

### 2.3. 3-Deazaneplanocin a (DZNep) Treatment of TKPTS

Cultured TKPTS cells at 50% confluence were serum-deprived overnight followed by treatments with 5 mg/mL Co-BSA or 5 mg/mL AGE-BSA, in the presence or absence of 5 μM EZH2 methyltransferase inhibitor 3-deazaneplanocin A (DZNep; Cayman Chemical, Ann Arbor, MI, USA) for 24 h.

### 2.4. RNA Isolation, Reverse Transcription, and qRT-PCR

Total RNA was isolated using the NucleoSpin^®^ Tissue Kit (Macherey-Nagel, Dueren, Germany) according to the manufacturer’s instructions. All cDNAs were prepared using 1 µg total RNA with the Promega Reverse Transcription system (Promega, Walldorf, Germany) and Oligo (dT)_15_ Primer. The mRNA expression was assayed by qRT-PCR using the qTOWER 2.2 Real-Time PCR Thermocycler (Analytik Jena AG, Jena, Germany). The gene-specific primers used for analyses were purchased from Invitrogen, Darmstadt, Germany and are listed in Table S1. For mRNA quantification, 1 µL of cDNA was subjected to qRT-PCR using 1 µM of each forward and reverse primer and ORA^TM^SEE qPCR Green ROX L Master mix (highQU GmbH, Kreichtal, Germany) according to the manufacturer’s instructions. The relative mRNA expression was quantified by qPCRsoft 4.1 software (Analytik Jena AG, Jena, Germany) and calculated using the ∆∆CT method [27]. The CT values of the gene of interest were normalized to housekeeping gene hypoxanthine-guanine-phosphorybosyl-transferase (*Hprt*) gene CT values (ΔCT). The gene expression ratio (R) was calculated relative to control as R = 2^(−ΔΔCT)^) [27].

### 2.5. Western Blotting

Treated TKPTS cells were lysed in 300 µL of cold Complete™ Lysis-M buffer supplemented with protease inhibitors (Roche Diagnostics GmbH, Mannheim, Germany). Cells were scraped, resuspended, and lysed on ice for 30 min. Afterwards lysates were centrifuged for 10 min at 13,000 rpm at 4 °C. To quantify the protein expression levels, a DeNovix DS-11FX Spectrophotometer (DeNovix Inc., Wilmington, DE, USA) was used. Protein lysates were separated on a 12% or 15% SDS-PAGE as suitable and were transferred on PVDF membrane by semi-dry transfer, as described elsewhere. All primary antibodies were incubated overnight at 4 °C. The next day, the membranes were washed 3 times 10 min each with 1xPBS solution, and the secondary antibodies were added for 2 h at room temperature. The primary and secondary antibodies used are listed in Table S2 and Table S3, respectively. Proteins were detected with ECL reagent (Revvity Health Sciences, Waltham, MA, USA) using the iBright™ FL1500 Imaging System (Thermo Fisher, Dreieich, Germany). Protein expressions were quantified using Fiji ImageJ-win64 software and were normalized to the housekeeping proteins TBP (TATA box binding protein), Vinculin, or Histone 3, as shown under the figure legends.

### 2.6. Immunofluorescence Staining of Cultured TKPTS Cells

Cells were cultured in Lab-Tek™ 4-well Permanox^®^ chambers (Thermo Fisher, Dreieich, Germany), serum-deprived and treated with Co-BSA or AGE-BSA for 24 h. Afterwards the cells were washed with PBS and fixed for 30 min at room temperature in PBS containing 3.7% Paraformaldehyde and 0.1% Triton X-100. Slides were next washed three times with PBS and nonspecific binding sites were blocked for 1 h in 5% BSA. Primary antibodies (listed in Table S2), diluted in blocking buffer, were applied overnight at 4 °C. The following day, the cells were washed 3 times 10 min each with PBS solution and subjected to an intermediate 30 min blocking step. Fluorescently labeled secondary antibodies (for details see Table S3) were incubated for 2 h at room temperature in the dark. For H3K27me3 antibody, which was directly conjugated to Alexa Fluor^®^ 647, the secondary antibody step was omitted, and the primary antibody was incubated overnight at 4 °C in the dark. After intensive washing with PBS, the nuclei were counterstained with DAPI (4′, 6-diamidino-2-phenylindole), and slides were mounted with Vectashield Vibrance^®^ antifade mounting medium (Vector Laboratories Inc., Newark, CA, USA). The images were acquired with the fluorescence microscope AxioImager 2 coupled with Zen 3.11 software (Carl Zeiss AG, Jena, Germany). Quantification was performed using Fiji ImageJ –win64 software by measuring the relative signal intensity, and data are presented graphically as box-and-whisker-dot plots, plotted using SigmaPlot v16 software (Systat, Frankfurt am Main, Germany). In addition we applied QuPath software (version 0.5.1; https://qupath.github.io/; accessed on 16 May 2025), using automated cell detection based on nuclear DAPI counterstaining and a custom groovy script and performed a second analysis of the intensity staining, where the percent of the positively stained nuclei was assayed relative to the total nuclei above the set threshold intensity. The threshold intensity is shown under the figure legends of the corresponding figures [28]. Cells were counted using a custom groovy script. The script is provided in the Supplementary Information.

### 2.7. Animals Studies

In this study, 20–25 week-old diabetic mice (*db/db*) and their non-diabetic littermates (control) were analyzed. The primary aim was to analyze the sex-related influence and effects of diabetes mellitus on the immunological detection of EZH2 protein expression and the levels of the repressive mark H3K27me3 in renal sections. All animal studies were approved by the local ethics committee (UKJ-17-024; approval date 6 December 2017) and were conducted in accordance with the German Animal Protection Law. The *db/db* diabetic mouse (strain: BKS.*Cg-Dock7m+/+ Leprdb*/J; Genetic Background C57BLKS/J) serves as a well-established animal model to study type 2 diabetes mellitus (T2DM) [24]. These mice have a spontaneous mutation in the Leptin receptor (*Lepr*) gene, which, in case of homozygous mutation, are characterized by obesity, insulin resistance, increased blood sugar, and the development of diabetic sequelae such as diabetic nephropathy. Notably, these mice develop diabetes as early as at the age of 6 weeks. Animals were purchased from Jackson Laboratory (Bar Harbor, ME, USA; Jax000642), breaded and housed under identical, standardized, pathogen-free conditions (SPF hygiene status) with a strict 12 h light/dark cycle. Mice were provided with standard laboratory chow diet and had unrestricted access to water at all times.

Genotyping was performed using DNA obtained from ear biopsies. For the analyses, the mice were divided into four groups based on their genotype and sex: (1.) non-diabetic males (control group; *Lepr^wt^*^/*wt*^), (2.) *db/db* diabetic males (*Lepr^db^*^/*db*^), (3.) non-diabetic females (control group; *Lep*r*^wt^*^/*wt*^), and (4.) *db/db* diabetic females (*Lepr^db^*^/*db*^). Animals were randomly assigned to experimental groups (with N = 3 animals per group; 12 animals in total) to avoid selection bias. No inclusion or exclusion criteria were set. Since this study was an experimental set used as a pilot experiment, no sample size calculation was performed.

### 2.8. Detection of H3K27me3 Levels in Proximal Tubules by Double Immunofluorescence Staining

The expression levels of EZH2 and H3K27me3 were further analyzed on 3 µm renal paraffin sections obtained from every single mouse across all four experimental groups. Kidney sections were prepared using a Rotary Microtome RM 2265 (Leica, Wetzlar, Germany). Slides were deparaffinized with xylene (Carl Roth, Karlsruhe, Germany), then rehydrated through descending alcohol series and subjected to heat-mediated antigen retrieval in citrate buffer (pH 6.0) for 30 min. After following thorough washing, the renal sections were blocked in 5% BSA in PBS for 1 h at RT. The protein levels of EZH2, H3K27me3, or γ-Glutamyl transferase (GGT1) were examined using primary antibodies listed in Table S2, followed by the fluorophore-labelled secondary antibodies detailed in Table S3.

The proximal tubules were identified by GGT1 staining, which serves as a specific marker for the proximal tubules [29]. The expression of EZH2 was evaluated throughout the entire tubular region of the kidney, while the tri-methylation of the repressive mark H3K27me3 was quantified in proximal tubules co-stained for both GGT1 and H3K27me3. The nuclei were counterstained with DAPI. Slides were then washed in PBS and mounted in Vectashield Vibrance^®^ antifade mounting medium (Vector Laboratories Inc., CA, USA). From each group 3 µm paraffin renal sections were stained with EZH2 or H3K27me3 specific antibodies. The following groups were subjected to analyses: 3× non-diabetic (wild-type WT) male mice; 3× *db/db* male mice; 3× non-diabetic (wild-type WT) female mice; 3× *db/db* female mice. From each staining of 8–10 fields at 400× magnifications of EZH2 or H3K27me3 stains, the staining intensities were analyzed with Fiji ImageJ software, and the averages of each field were statistically analyzed. Generating thus from N = 3 mice per group, × 8–10 images were analyzed finally for n = 28–30 staining intensities per group of each staining for EZH2 or H3K27me3, respectively. Images were acquired using a fluorescence microscope and AxioImager 2 and Zen 3.11 software (Carl Zeiss AG, Jena, Germany). Staining intensities were quantified using Fiji ImageJ software and are graphically presented in box-and-whisker-dot plot. Investigators were blinded to the different groups during data collection and analysis. For additional quantification, cells with signal intensity above a certain threshold were counted using QuPath (version 0.5.1; https://qupath.github.io/; accessed on 16 May 2025) using automated cell detection based on nuclear DAPI counterstaining and a custom groovy script. The script is provided in the Supplementary Methods.

### 2.9. Statistical Analyses

Statistical analyses were performed using SigmaPlot v16 software (Systat, Frankfurt am Main, Germany). The data are presented graphically as box-and-whisker-dot plots, plotted using SigmaPlot v16 software. For multiple comparison, one-way AVOVA followed by the Holm–Sidak method was used, if normality was found. To compare two groups, the Mann–Whitney U test was applied. Multiple group comparison was performed with the Mann–Whitney U test for hypothesis-driven pairwise analyses, while Kruskal–Wallis results are provided for global comparison among all groups. Outliers were checked using box plots and included only if biologically justified. A *p*-value < 0.05 was considered statistically significant. * *p* < 0.05, ** *p* < 0.01, *** *p* < 0.001

## 3. Results

### 3.1. Expression of Receptor for Advanced Glycation End Products (RAGEs) in TKPTS Cells

**Figure 1:**
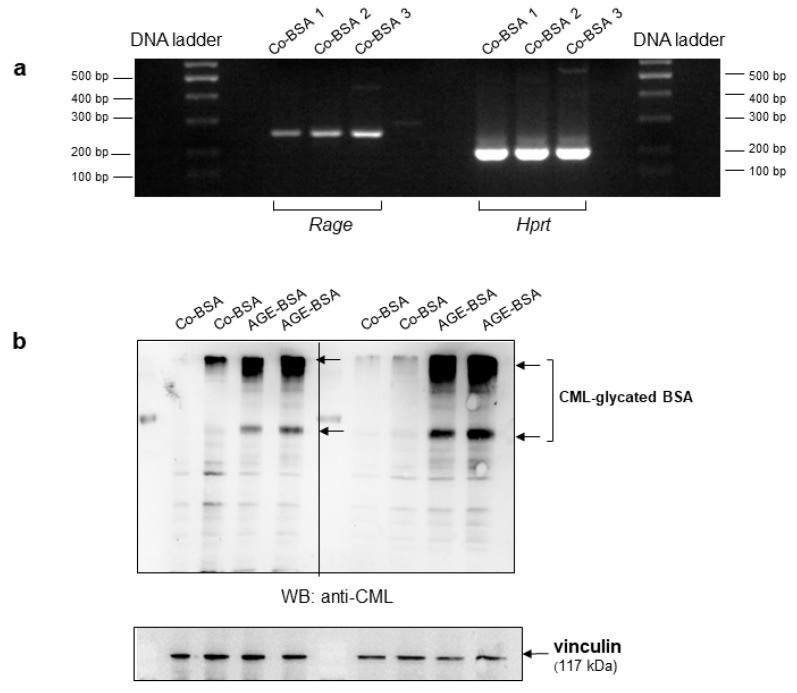
Expression of receptor for advanced glycated end products (RAGEs) in TKPTS cells and detection of N^ε^-carboxy-methyl lysine modification in protein lysates from glycated-BSA treated cells. (**a**) Detection of RAGE by semi-quantitative PCR from three independent Co-BSA treated TKPTS cells on 2% agarose gel. Representative image of PCR amplification of *Rage* and *Hprt* (hypoxanthine-guanine-phosphoribosyl-transferase) is shown. The DNA ladder and corresponding PCR bands for *Rage* and the loading control *Hprt* are labeled. (**b**) Detection of N^ε^-carboxy-methyl lysine (CML) by Western blot in total protein lysates from TKPTS cells incubated for 24 h with Co-BSA or AGE-BSA. Detection of CML in four different protein lysates per treatment are shown. The arrows show the CML-glycated BSA. Vinculin expression was analyzed as a loading control after membrane stripping.

Previous studies, including our own, have reported the expression of the receptor for advanced glycated end products (RAGEs) in differentiated glomerular podocyte cell lines [30,31,32]. RAGE expression has also been described in BUMTP cells (a mouse proximal tubular derived cell line) [33,34]. In the present study, to address the function of AGEs, we used a widely studied proximal tubular cell line termed TKPTS and evaluated whether RAGE is expressed in the TKPTS cells. Therefore, we subjected cDNAs originating from TKPTS, treated with control-BSA to semi-quantitative PCR analyses. Our data revealed that a band corresponding to the expected size for a *Rage* DNA-fragment was amplified; in parallel reactions the expression of *Hprt* (hypoxanthine-guanine-phosphoribosyl-transferase) as a housekeeping gene was also analyzed (Figure 1a).

We next examined the uptake of AGE-BSA into TKPTS cells using Western blot analyses. The presence of N^ε^-carboxy-methyl lysine (CML), a common glycation modification, was assessed in total cell lysates. CML was detected in protein lysates from AGE-BSA treated cells but not in Co-BSA treated cells (Figure 1b). We identified in the total protein lysates a primary band corresponding to the CML-modified BSA (shown with the arrows on the image) and several higher molecular weight bands, suggesting cross-linked complexes of glycated-BSA. Therefore, RAGE is expressed in TKPTS cells, and glycated-BSA can bind to its receptor and distribute the pathological signals into TKPTS cells.

### 3.2. AGE-BSA Reduces the Expression of EZH2 in Proximal Tubular TKPTS Cells

**Figure 2:**
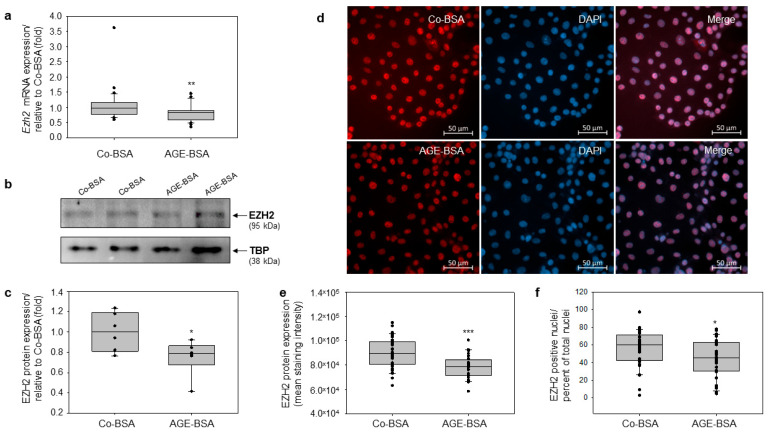
Glycated-BSA diminished the expression of EZH2 in murine TKPTs cells. The cells were treated for 24 h with Co-BSA or AGE-BSA followed by RNA isolation, cDNA synthesis, and qRT-PCR analyses. (**a**) Real-time PCR analyses of *Ezh2* mRNA expression. Treatment with 5 mg/mL AGE-BSA for 24 h significantly reduced *Ezh2* mRNA levels in TKPTs relative to 5 mg/mL Co-BSA treated cells (n = 30). (**b**) Western blot analysis of EZH2 protein expression. Representative images of EZH2 and TATA box binding protein (TBP) as a loading control are shown. (**c**) Western blot densitometry. EZH2 protein expression was normalized to TBP expression and presented in fold relative to Co-BSA EZH2 expression levels (n = 6). (**d**) Immunofluorescence staining of EZH2 in TKPTs cells treated for 24 h with Co-BSA or AGE-BSA. The nuclei were counterstained with DAPI. Representative images of single channel detection and merge images are shown. Scale bar: 50 µm. (**e**) Densitometry analysis of the EZH2 staining intensity for Co-BSA and AGE-BSA. Three independent staining experiments were analyzed. From each staining 10 fields corresponding to 400x magnifications were measured. n = 30 per treatment. (**f**) Analysis of EZH2-positive nuclei presented as percent of total nuclei. EZH2 staining analysis was performed using QuPath by quantifying cells showing signal intensities above a defined threshold of 7000, expressed as percent of total cell number. Three independent staining experiments were analyzed. From each staining 10 fields corresponding to 400× magnifications were measured. n = 30 per treatment. The Mann–Whitney U test was used for two-group comparison. * *p* < 0.05, ** *p* < 0.01; *** *p* < 0.001 versus Co-BSA.

Evidence shows that hyperglycemia promotes the formation of advanced glycation end products (AGEs) through non-enzymatic reactions [5,6]. AGEs contribute to the progression of diabetic disease and the development of diabetic complications [35]. We previously reported that AGEs, via their receptor RAGE, can modulate the expression of many genes [30,35]. The enhancer of zeste homolog 2 (EZH2), a histone H3 lysine K27 (H3K27) methyltransferase, plays a key role in the epigenetic regulation [11,12]. We recently reported that AGE-BSA reduced the expression of EZH2 in the differentiated murine podocyte cell line [18]. However, whether AGEs influenced the EZH2 expression in proximal tubules has not been investigated. To address this, TKPTS cells were treated for 24 h with either control-BSA (Co-BSA) or glycated-BSA (AGE-BSA). Quantitative RT-PCR analyses revealed modest but significant reduction of *Ezh2* mRNA levels in AGE-BSA treated TKPTS cells compared with Co-BSA treated controls (Figure 2a). Furthermore, Western blot studies confirmed that AGE-BSA also significantly reduced EZH2 protein abundance in TKPTS cells (Figure 2b,c). We also assayed the influence of AGE-BSA on EZH2 protein expression via immunofluorescence detection. Our analyses showed a nuclear localization of EZH2 protein (Figure 2d). Densitometry analyses showed a significantly decreased EZH2 staining intensity in AGE-BSA treated cells relative to Co-BSA treatments (Figure 2e). Counting of EZH2-positive nuclei compared to the total cell number revealed a similar pattern, with significantly less cells exceeding the threshold in AGE-BSA treated cells. (Figure 2f).

### 3.3. AGE-BSA Reduces Histone H3K27 Tri-Methylation Levels in TKPTS Cells

**Figure 3:**
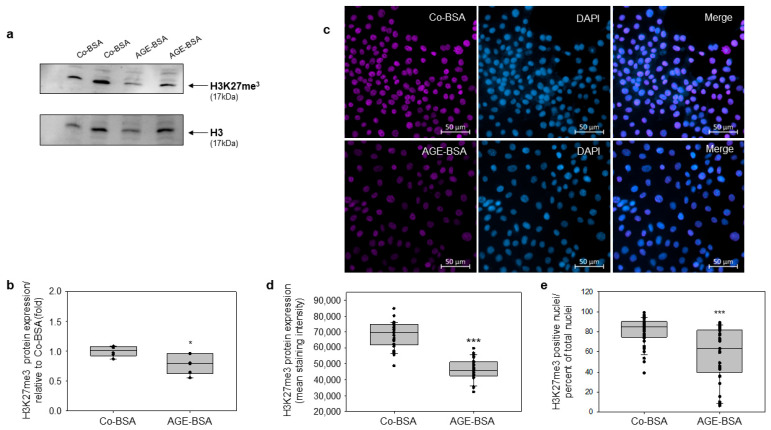
Glycated AGE-BSA contributes to reduced expression of the repressive mark H3K27me3 in TKPTs cells. The cells were treated for 24 h with Co-BSA or AGE-BSA and H3K27me3 protein levels were detected in total cell lysates. (**a**) Detection of the H3K27 tri-methylation levels by Western blotting. Representative images of H3K27me3 (up) and H3 protein expression (down) as loading control in total cell lysates are shown. Exposure of the cells to AGE-BSA for 24 h significantly reduced the H3K27me3 levels in TKPTs relative to Co-BSA treated cells. (**b**) Western blot densitometry. H3K27me3 protein levels was normalized to H3 expression and presented in fold relative to Co-BSA (n = 6). (**c**) Detection of H3K27me3 tri-methylation in TKPTs cells treated with Co-BSA or AGE-BSA by immunofluorescence staining. The nuclei were counterstained with DAPI. Representative images of single channel detection as well as merge images are shown. Scale bar: 50 µM. (**d**) Densitometry analysis of the H3K27me3 staining intensity for Co-BSA and AGE-BSA. Three independent staining experiments were subjected to analysis. From each staining 10 fields corresponding to 400× magnifications were analyzed. n = 30 per treatment. (**e**) Analysis of H3K27me3-positive nuclei presented as percent of total nuclei. H3K27me3 staining analysis was performed using QuPath by quantifying cells showing signal intensities above a defined threshold of 4000, expressed as a percent of the total cell number. Three independent staining experiments were analyzed. From each staining 10 fields corresponding to 400× magnifications were measured. n = 30 per treatment. Mann–Whitney U test was used for two-group comparison. * *p* < 0.05, *** *p* < 0.001 versus Co-BSA.

The repressive mark H3K27me3 is an epigenetic modification characteristic for repression of the gene expression and gene transcription [36,37]. Recent studies have shown that the alteration of the H3K27me3 is observed in diabetes as well as in diabetic kidney disease (DKD) [18,38]. The EZH2 methyltransferase catalyzes the mono-, di-, and tri-methylation of lysine-27 on histone H3 [39]. Because EZH2 protein expression was attenuated in AGE-BSA treated TKPTS cells, we examined whether this treatment also affects the repressive histone 3 mark H3K27me3 methylation. We used a Western blot and immunostaining approach to study the H3K27me3 levels in TKPTS cells. Our data from protein analyses showed that H3K27me3 was significantly reduced upon application of AGE-BSA to the cells (Figure 3a,b). Furthermore, detection of H3K27me3 by immunofluorescence stain unveiled that cells treated with AGE-BSA have a significantly decreased level of H3K27me3 than the cells exposed to control-BSA (Figure 3c,d). Counting of H3K27me3-positive nuclei compared to the total cell number revealed a similar pattern, with a significantly decreased percentage of positive nuclei in AGE-BSA treated cells. (Figure 3e). Thus, AGE-BSA via reduction of EZH2 expression mediates epigenetic alteration on the repressive mark H3K27me3 in TKPTS cells.

### 3.4. Treatment of TKPTS Cells with AGE-BSA Does Not Affect the Expression of Nuclear Inhibitor of PP1 Phosphatase (NIPP1)

**Figure 4:**
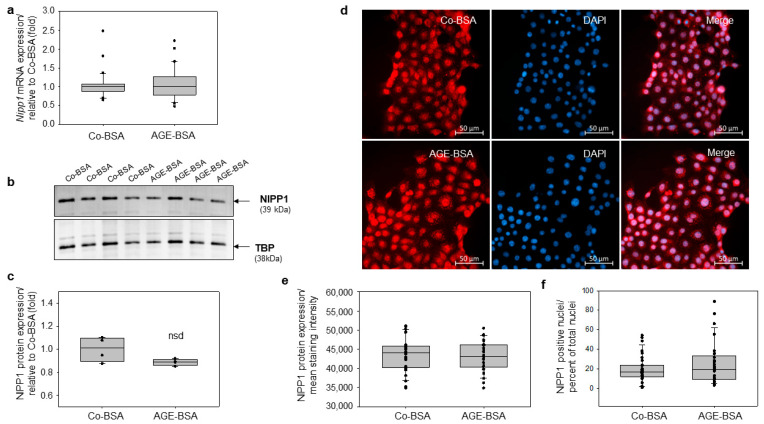
Glycated-BSA did not affect the expression of NIPP1 in murine TKPTs cells. (**a**) Real-time PCR analyses of *Nipp1* mRNA expression. Treatment of TKPTS cells with 5 mg/mL AGE-BSA for 24 did not affect *Nipp1* mRNA levels (n = 30). (**b**) Representative images of NIPP1 protein expressions detected in total cell lysates. TATA-Binding Protein (TBP) expression was detected as loading control. (**c**) Western blot densitometry. NIPP1 protein expression was normalized to TBP expression and presented in fold relative to Co-BSA treated samples. (**d**) Immunofluorescence staining of NIPP1 in TKPTs cells treated for 24 with Co-BSA or AGE-BSA. The nuclei were counterstained with DAPI. Representative images are shown. Scale bar: 50 µM. (**e**) NIPP1 protein expression was measured with Fiji ImageJ Software and is shown as mean staining intensity in Co-BSA and AGE-BSA treated cells. Three independent staining experiments were analyzed. From each staining 10 fields corresponding to 400× magnifications were analyzed. n = 30 per treatment. (**f**) Analysis of NIPP1-positive nuclei presented as percent of total nuclei. NIPP1 staining analysis was performed using QuPath by quantifying cells showing signal intensities above a defined threshold of 9000, expressed as a ratio relative to the total cell number. Three independent staining experiments were analyzed. From each staining 10 fields corresponding to 400× magnifications were measured. n = 30 per treatment. The Mann–Whitney U test was used for two-group comparison.

Previous studies have shown that glycated-BSA reduced the *Nipp1* mRNA and protein expression in differentiated podocytes compared with control BSA [18,40]. Furthermore, NIPP1 was an important factor involved in the regulation of the EZH2 expression in podocytes, and its decreased expression in AGE-BSA treated differentiated podocytes was associated with diminished EZH2 and H3K27me3 protein levels [18]. In this context, we investigated whether glycated-BSA can affect the expression of NIPP1 in TKPTS cells. Our data revealed that neither mRNA nor the protein expression of NIPP1 were affected significantly by the treatment of the cells with glycated-BSA (Figure 4a–c). These findings were confirmed by the immunofluorescent detection of NIPP1 expression in TKPTS cells. Our findings revealed that the treatment with AGE-BSA did not significantly alter the NIPP1 protein levels (Figure 4d,e). In addition, no change was detected in an additional analysis counting NIPP1-positive nuclei relative to the total cell number. (Figure 4f).

### 3.5. Influence of AGE-BSA on the Expression of Genes Involved in Proximal Tubular Cell Damage

**Figure 5:**
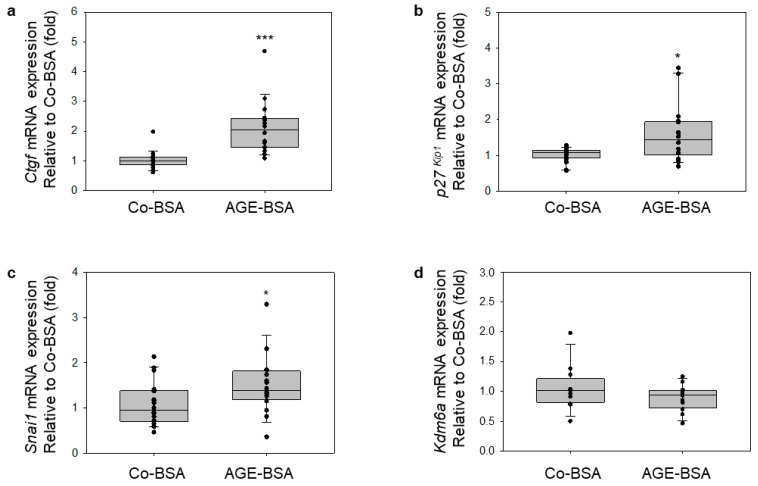
Glycated-BSA in TKPTS cells induced the expression of genes associated with the proximal tubular damage in diabetic kidneys. Real-time PCR analyses. Treatment of the TKPTS cells with glycated-BSA (AGE-BSA) significantly increases the expression of genes, contributing to the progression of DKD and renal injury as *Ctgf*, *p27^Kip1^*, and *Snai1*. (**a**) qRT-PCR analyses of connective tissue growth factor *Ctgf* expression, n = 18, *** *p* < 0.001 vs. Co-BSA. (**b**) Quantification of *p27^Kip1^*, mRNA expression by qRT-PCR, n = 18 per treatment. * *p* < 0.05 vs. Co-BSA. (**c**) qRT-PCR analyses of *Snai1* mRNA expression, n = 16–18, * *p* < 0.05 vs. Co-BSA. (**d**) Evaluation of *Kdm6a* gene expression by real-time PCR. The ubiquitously transcribed tetratricopeptide repeat X chromosome (UTX) is H3K27me3 demethylase encoded by the *Kdm6a* gene. n = 12, nsd in the *Kdm6a* mRNA expression levels between Co-BSA and AGE-BSA in TKPTS cells. Mann–Whitney U test was used for two-group comparison.

To examine whether AGE-BSA treatment alters the expression of known genes associated with proximal tubular cell damage in diabetic kidney disease and EMT progression [41], we measured mRNA levels of connective tissue growth factor (*Ctgf*), *p27^Kip1^*, and *Snai1* genes in TKPTS cells. The expression of all three genes was significantly induced after AGE-BSA treatment compared with Co-BSA controls (Figure 5a–c). Similar to our observations in differentiated podocytes, glycated-BSA modulates the expression of these genes, which protein products are well-known to be associated with the development of diabetic nephropathy.

These findings suggest that AGE-BSA, or AGEs in general, in proximal tubular cells may contribute to (I) the onset of renal fibrosis through *Snai1* induction [42,43]; (II) activation of genes involved in the endothelial-to-mesenchymal transition (EMT) via elevated *Ctgf* mRNA expression [44]; and (III) proximal tubular cells hypertrophy via *p27^Kip1^* [45], a mechanism previously linked to elevated glucose or increased levels of Angiotensin II.

We also investigated whether AGE-BSA treatment affect the expression of UTX, an enzyme that regulates the methylation state of H3K27me3 via its demethylase enzymatic activity [46] and is encoded by the *Kdm6a* gene [46]. Our results revealed that treatment of TKPTS cells with glycated-BSA for 24 h did not significantly change *Kdm6a* mRNA levels compared to Co-BSA treated cells (Figure 5d).

### 3.6. Application of DZNep, an EZH2 Inhibitor, Reduces H3K27me3 in TKPTS Cells

It has been previously shown that 3-Deazaneplanocin A (DZNep), an EZH2 inhibitor, originally identified as a S-adenosylhomocysteine hydrolase inhibitor, also indirectly inhibits the histone-methyltransferase activity of the EZH2 protein and specifically decreases the H3K27 methylation without affecting the H3 K9 methylation [47,48].

**Figure 6:**
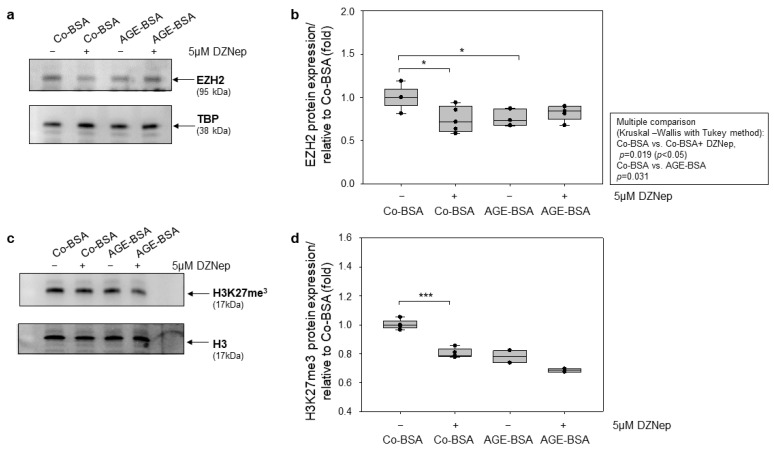
Pharmacological inhibition of EZH2 with 3-Deazaneplanocin A (DZNep) suppressed more effectively the repressive mark H3K27 tri-methylation than EZH2 protein expression in TKPTS cells. (**a**) Western blot analyses of EZH2 expression. Representative images of EZH2 proteins expressions detected in total cell lysates. TBP protein expression was detected as a loading control. (**b**) Western blot densitometry. EZH2 protein expression was normalized to the corresponding loading control and is presented in fold relative to Co-BSA treated samples. n = 5-5-5-5; * *p* < 0.05. (**c**) Western blot analyses of H3K27me3 levels. Representative image from H3K27me3 tri-methylation in total cell lysates. Histone 3 protein expression was detected as loading control. (**d**) Western blot densitometry. H3K27me3 protein level was normalized to the corresponding loading control and is presented in fold relative to Co-BSA treated samples. n = 5-5-2-2. Statistical analyses were only conducted for n ≥ 3; the Mann–Whitney U test was used for two-group comparison and is presented with brackets above the box plots and marked with significance stars. For multiple group comparison, the Kruskal–Wallis test was performed, and the results are shown in the table next to the figure. * *p* < 0.05; *** *p* < 0.001.

To evaluate the potential effect of pharmacological EZH2 inhibition, we treated TKPTS cells with DZNep and assessed EZH2 and H3K27me3 levels. We first examined whether DZNep treatment affects EZH2 protein expression in TKPTS cells. Western blot analyses revealed that EZH2 inhibitor significantly reduced EZH2 protein expression in Co-BSA plus DZNep treated cells relative to Co-BSA alone, but did not further decrease EZH2 expression in AGE-BSA plus DZNep compared with AGE-BSA alone (Figure 6a,b). We further examined whether DZNep treatment may affect the H3K27 tri-methylation. We found that inhibition of EZH2 methyltransferase activity by application of 5 µM DZNep for 24 h to TKPTS cells significantly reduced the repressive mark H3K27 tri-methylation in Co-BSA plus 5 µM DZNep treated cells compared with Co-BSA alone (Figure 6c,d). Although we just show a qualitative, mechanistic observation, due to insufficient sample size, we observe a tendency of further decreased levels of H3K27me3 in AGE-BSA plus 5 µM DZNep compared to AGE-BSA. This observation indicates that DZNep primarily inhibits EZH2 methyltransferase activity rather than affecting its protein expression.

### 3.7. Inhibition of EZH2 Transmethylase Activity by DZNep Increases the Expression of Genes Involved in Kidney Injury

**Figure 7:**
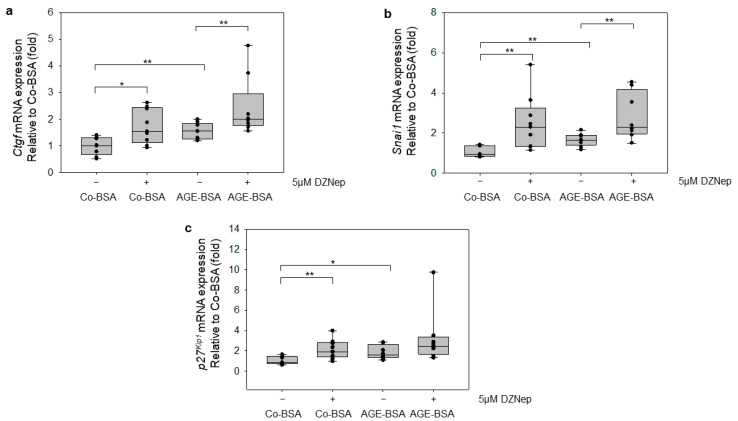
Pharmacological inhibition of EZH2 with DZNep induced *Ctgf*, *p27^Kip1^*, and *Snai1* mRNA expression in TKPTs cells. Real-time PCR analyses. Treatment of the TKPTS cells with 5µM DZNep significantly increases the expression of genes contributing to the progression of DKD and renal injury (**a**) qRT-PCR analyses of *Ctgf* (connective tissue growth factor) mRNA expression, n = 9-9-9-9. * *p* < 0.05, ** *p* < 0.01. (**b**) qRT-PCR analyses of *Snai1* mRNA expression, n = 7-9-9-8 per treatment. ** p < 0.01. (**c**) Quantification of *p27^Kip11^* mRNA expression by qRT-PCR, n = 7-9-9-8 per treatment. * *p* < 0.05, ** *p* < 0.01. The paired box plots to which the Mann–Whitney U test applies are connected with brackets, and the corresponding significance is shown with significance stars.

We next analyzed whether applying DZNep to TKPTS cells affects the expression of genes related to renal damage *Ctgf*, *Snai1*, and *p27^Kip1^* [49]. The cells were treated with Co-BSA or AGE-BSA in the presence or absence of 5 µM DZNep for 24 h. Pharmacological inhibition of EZH2 activity significantly increased the mRNA expression of *Ctgf* (Figure 7a), *Snai1* (Figure 7b), and *p27^Kip1^* (Figure 7c) compared with Co-BSA treated cells. This observation suggests that reduced methylation of the repressive mark H3K27me3 may activate the expression of these genes, thereby promoting proximal tubule injury. Furthermore, a co-treatment with 5 µM DZNep and glycated-BSA further enhanced the expression of *Ctgf* and *Snai1* relative to AGE-BSA alone, whereas the *p27^Kip1^* mRNA expression was higher than in AGE-BSA alone but did not reach statistical significance levels.

### 3.8. Evaluation of Sex Differences in EZH2 Protein Expression in Renal Sections from Diabetic Mice

Previous studies from our lab have shown that EZH2 expression is decreased in glomerular podocytes of diabetic male mice compared with healthy controls [18]. However, female mice were not included in the evaluation of EZH2 expression in glomerular podocytes [18].

**Figure 8:**
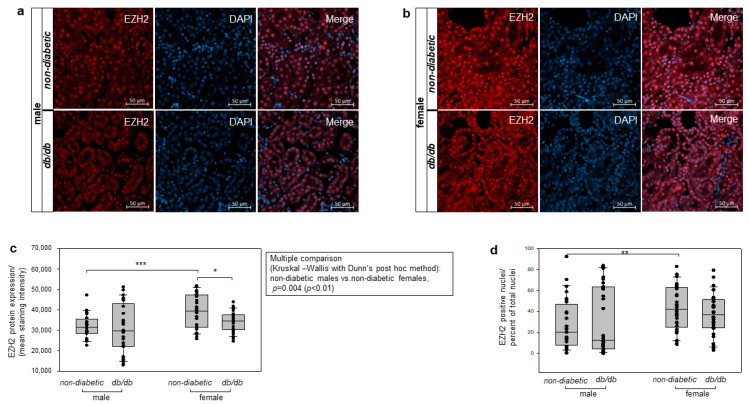
Detection of EZH2 protein expression in the tubular region of non-diabetic and diabetic mice. Sex-specific analysis. (**a**) Protein staining of EZH2 was assessed by immunofluorescence in renal sections from non-diabetic and diabetic male mice. Nuclei were counterstained with DAPI. Scale bar: 50 µm. Representative images show EZH2 staining (red), DAPI detection (blue), and merge channels. (**b**) Immunofluorescent detection of EZH2 expression in renal sections of non-diabetic and diabetic female mice. Nuclei were visualized with DAPI. Scale bar: 50 µm. Representative images show EZH2 (red), DAPI (blue), and merge channels. (**c**) Densitometry analysis of EZH2 staining intensity across the entire renal tubule region in non-diabetic and *db/db* male and female mice. The EZH2 protein expression was evaluated by Fiji ImageJ software and is graphically presented in a box-and-whisker-dot plot. n = 3 mice per group; 10 fields per mouse at 400× magnifications were measured; n = 30 per group. (**d**) Analysis of EZH2-positive nuclei presented as percent of total nuclei. EZH2 staining analysis was performed using QuPath by quantifying cells showing signal intensities above a defined threshold of 7000, expressed as a ratio relative to the total cell number across the entire renal tubule region in non-diabetic and *db/db* male and female mice. n = 3 mice per group; 10 fields per mouse at 400× magnifications were measured; n = 30 per group. Mann–Whitney U test was used for two-group comparison and is presented with brackets above the box plots and marked with significance stars. For multiple group comparison, the Kruskal–Wallis test was performed, and the results are depicted in the table next to the figure. * *p* < 0.05, ** *p* < 0.01, *** *p* < 0.001; EZH2, enhancer of zeste homolog 2; DAPI, 4′,6-diamidino-2-phenylindole.

In the present study, we examine the tubular EZH2 expression in 24-week-old male and female diabetic and age-matched non-diabetic mice. The diabetic mice were obese (body weight: males 50.7 ± 4.3 g; females 47.4 ± 6.2 g) and exhibited hyperglycemia (fasting blood glucose: males 12.8 ± 11.6 mmol/L; females 9 ± 3.3 mmol/L). We evaluated the EZH2 expression across the entire tubular region of all four mice groups using immunofluorescent staining of renal sections from non-diabetic and diabetic male (Figure 8a) and female mice (Figure 8b). Our data showed that there was no significant difference in the tubular EZH2 expression between non-diabetic and diabetic male mice (Figure 8c). In contrast, we observed a significant reduction in EZH2 protein expression in diabetic females compared to non-diabetic females (Figure 8c).

Moreover, staining-intensity measurements showed significantly lower EZH2 expression in non-diabetic male mice relative to age-matched non-diabetic females. Interestingly, the EZH2 expression in healthy male mice was also significantly lower than in diabetic female mice (Figure 8c), whereas no significant difference in EZH2 expression between diabetic male and female mice was observed (Figure 8c). Counting of EZH2-positive nuclei across the renal tubule region compared to the total cell number revealed a similar result. Non-diabetic females showed a significantly higher number of EZH2-positive nuclei than non-diabetic males (Figure 8d).

### 3.9. Evaluation of Sex Differences in H3K27me3 Protein Expression in Renal Sections from Diabetic Mice

We recently analyzed the tri-methylation state of H3 lysine 27 in glomerular podocytes of non-diabetic and *db/db* mice [18]. Our data revealed that H3K27me3 is significantly decreased in *db/db* mice, a well-established animal model of type 2 diabetes mellitus [24,50] compared with age-matched healthy controls [18]. As mentioned above the referred study included only male diabetic and non-diabetic mice [18].

In the present work, we aimed to determine whether sex influences the repressive mark H3K27me3 in proximal tubules of diabetic and non-diabetic mice. To specifically asses the proximal tubular levels of histone 3 K27 tri-methylation, we performed double immunofluorescent staining of H3K27me3 and γ-Glutamyl transpeptidase (GGT1), a well-characterized maker for renal proximal tubular cells [29,51]. The H3K27me3 staining was only quantified in GGT1-positive tubules.

**Figure 9:**
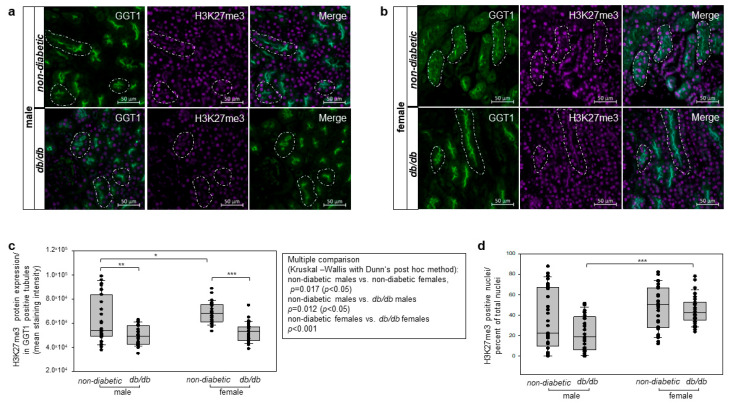
Immunofluorescent detection of H3K27me3 tri-methylation levels in proximal tubule of non-diabetic and diabetic mice. Influence of a sex-specificity. (**a**) Detection of repressive histone 3 mark H3K27me3 and GGT1 in proximal tubular cells by double immunofluorescent staining in renal sections of non-diabetic and diabetic male mice. Some proximal tubules on the images are surrounded by white dashed ovals shown in all channels. Representative images of GGT1 (green), H3K27me3 (purple), and merged channels are shown. n = 3 mice per group were analyzed. Scale bar: 50 µm. (**b**) Analysis of H3K27me3 and GGT1 protein expression by double immunofluorescence stain in renal sections from non-diabetic and diabetic female animals. Similarly as in (**a**), the GGT1-positive staining corresponds to the proximal tubule, and the double positive GGT1 and H3K27me3 tubules show the methylation levels of H3K27me3 in the kidney proximal tubules. Some proximal tubules on the images are surrounded by white dashed ovals. Representative images of GGT1 (green), H3K27me3 (purple), and merge channels are shown. n = 3 mice per group were analyzed. Scale bar: 50 µm. (**c**) Quantification of H3K27me3 methylation levels in proximal tubules detected in non-diabetic and *db/db* male and female mice. The proximal tubule-specific detection of H3K27me was measured by Fiji ImageJ software in GGT1 and H3K27me3 double positive tubules and is graphically presented in a box-and-whisker-dot plot. n = 3 mice per group were subjected to analyses; 10 fields per mouse at 400× magnifications were measured; n = 30 per group. (**d**) Analysis of H3K27me3-positive nuclei presented as percent of total nuclei. H3K27me3 staining analysis was performed using QuPath by quantifying cells showing signal intensities above a defined threshold of 4000, expressed as a ratio relative to the total cell number in proximal tubules in non-diabetic and *db/db* male and female mice. n = 3 mice per group; 10 fields per mouse at 400× magnifications were measured; n = 30 per group. Mann–Whitney U test was used for two-group comparison and is presented with brackets above the box plots and marked with significance stars. For multiple group comparison, the Kruskal–Wallis test was performed, and the results are depicted in the table next to the figure. ** *p* < 0.01 versus non-diabetic animals. *** *p* < 0.001 versus non-diabetic animals. H3K27 me3, histone 3 K27 tri-methylation; GGT1, γ-Glutamyl transpeptidase; * *p* < 0.05, ** *p* < 0.01, *** *p* < 0.001.

Our results depicted significantly reduced levels of H3K27me3 in proximal tubules of diabetic mice in both sexes (Figure 9a–c) compared with non-diabetic age-matched controls (Figure 9a–c). Furthermore, non-diabetic females exhibited significantly higher levels of the repressive mark H3K27me3 staining intensity than non-diabetic males (Figure 9c). Nevertheless, no significant sex-dependent difference was detected in H3K27me3 levels between diabetic male and female mice. Further analysis based on the quantification of H3K27me3-positive nuclei further promotes sex-dependent differences, with significantly higher H3K27me3 levels in diabetic females compared to diabetic males (Figure 9d). All channel images, including GGT1, H3K27me3, and DAPI, and their merge composite from Figure 9a,b, are provided as Supplementary Figures S1 and S2 for male and female mice, respectively.

These findings indicate that diabetic conditions are associated with diminished levels of the repressive histone 3 mark H3K27me3, which may contribute to increased transcription of genes involved in the pathogenesis of diabetic kidney disease.

## 4. Discussion

Advanced glycated end products (AGEs) are a diverse group of glycated proteins, lipids, or nucleic acids that result from non-enzymatic glycation in the course of uncontrolled hyperglycemia [52]. Ageing also promotes the chronic accumulation of AGEs and their deposition in renal tissues, contributing to pathophysiological age-dependent renal damage [1,52,53,54]. Some researchers have even suggested that AGEs are playing a more critical role in microvascular complication in diabetes than hyperglycemia itself [8].

Considerable attention has been focused on whether epigenetic alterations observed in diabetes mellitus type 2 are initiated early in the diabetic disease and how they relate to the so-called metabolic memory phenomenon. Numerous epigenetic changes including DNA methylation, histone methylation, histone acetylation, and the accumulation of long non-coding RNA [55] or miRNA [56,57,58] have been reported in diabetes [38,59]. Metabolic memory acts as a cellular “fingerprint”, persisting even when factors such as increased glucose levels, hypertension, or the accumulation of inflammatory cytokines are controlled [60]. Chilelli et al. [8] proposed that AGEs represent a kind of “micro-metabolic memory” because they remain in the cells far longer than transient hyperglycemia [8].

It has been shown that histone posttranslational modifications, including methylation, demethylation, acetylation, and deacetylation, regulate chromatin structure and transcription factor accessibility [61,62]. The enhancer of zeste homologue2 (EZH2), the main catalytic subunit of polycomb-repressive complex 2 (PCR2), specifically mediates H3K27 tri-methylation [63], thus silencing gene expression [62,63].

Recently, we showed that AGEs induced epigenetic alterations on the repressive mark H3K27me3 via reduced expression of the complex between nuclear inhibitor of PP1 phosphatase (NIPP1) and enhancer of zeste homolog 2 (EZH2) [18]. Based from our findings from podocytes, we explored whether AGEs similarly mediate epigenetic modifications in proximal tubular cells using TKPTS cells.

Our current data show that TKPTS cells expressed RAGEs, and the most common AGE modification N^ε^-carboxy-methyl lysine (CML) [52] was detected in lysates treated with glycated-BSA but not in Co-BSA, suggesting that AGEs can initiate pathological signals in proximal tubular cells via RAGEs. Previously we observed that glycated-BSA, diabetes, and hypoxia reduced EZH2 expression in differentiated podocytes and renal glomeruli [18,64], while in human aortic endothelial cells hyperglycemia induced EZH2 expression and increase the repressive mark H3K27me3, thus contributing to endothelial-to-mesenchymal transition (endoMT) [65,66]. Thakar et al. [66] also reported that in human umbilical vein endothelial cells (HUVECs), glucose fluctuation rather than stable high glucose levels induced EZH2 expression and H3K27me3, thus leading to Krueppel-like Factor 2 (KLF2) repression and endothelial inflammation in diabetes mellitus [66]. Inversely, in GDM-HUVECs isolated from gestational diabetes mellitus-affected individuals, Floris et al. [67] found that upregulation of miR-101 in these cells was associated with decreased EZH2 and H3K27me3 levels [67]. Furthermore, upregulation of miR-124 was reported to promote β-cells apoptosis by reduced EZH2 expression and de-repression of H3K27me3 [23].

Here, we show that treatment of TKPTS cells with glycated-BSA significantly decreased EZH2 mRNA and protein expression. Unlike podocytes [18], NIPP1 expression was unaffected by AGE-BSA treatment in TKPTS cells, suggesting a distinct and cell-type-specific mechanism of AGE-mediated epigenetic alteration in proximal tubular cells. The reduced EZH2 expression was associated with a decreased repressive mark H3K27me3. Consequently, we detected that glycated-BSA treatment significantly induced mRNA expression of *Snai1*, *Ctgf,* and *p27^Kip1^*. This revealed that AGEs through epigenetic alteration of H3K27me3 in proximal tubular cells may contribute to (I) the onset of renal fibrosis via *Snai1* induction [42,43]; (II) activation of genes involved in the endothelial-to-mesenchymal transition by elevating *Ctgf* mRNA expression [44,68]; and (III) proximal tubular cell hypertrophy via *p27^Kip1^* [45,69,70], mechanisms previously linked to elevated glucose or blood pressure. Our data is also in agreement with a recent report on mesangial cells, where the authors found that high glucose-induced TGF-β1 was associated with reduced EZH2 expression and dysregulation of H3K27me3 at the CTGF promoter [68].

Pharmacological inhibition of EZH2 activity by application of the 3-Deazaneplanocin A (DZNep) in TKPTS cells confirmed the functional role of EZH2. DZNep significantly reduced the H3K27me3 tri-methylation in Co-BSA treated cells and further decreased H3K27me3 in AGE-BSA treated cells. DZNep treatment also induced the *Ctgf*, *Snai1*, and *p27^Kip1^* mNA expression. Thus, our data highlight the protective role of EZH2 and H3K27me3 in proximal tubular cells against initiation of EMT, renal fibrosis, tubule interstitial fibrosis, and tubular hypertrophy. Furthermore, these findings extend our prior observation in podocytes, demonstrating the AGE-induced suppression of EZH2 contributes to epigenetic reprogramming and gene expression changes relevant to diabetic kidney disease.

Although sex-differences in diabetes and diabetic nephropathy have gained increasing attention during the recent years, the role of sex in the development and progression of diabetic kidney disease remains incompletely understood [71]. Recently, employing various approaches, we have shown that sex, sex hormones, and diabetic conditions regulate the expression of TGF-β1 through a complex crosstalk mechanism [25]. This regulation contributes to the progression of renal fibrosis and DKD by modulating the expression of pro-fibrotic genes such as *Ctgf* [25].

Despite the growing body of evidence demonstrating that epigenetic alteration plays a causal role in health and disease, the sex-specific influence in this process has been not at all or very little investigated. Therefore, our study contributes to a better understanding of the sex-dependent influence on EZH2 and H3K27me3 levels in renal tubules of non-diabetic and *db/db* mice, a model of type 2 diabetes [24]. Typical characteristics of the mouse model used, such as the degree of proteinuria and renal immunohistology findings (fibronectin and collagen I), have been described in detail in several previous studies from our group [25,41]. Interestingly, sex-specific analyses in renal tubules revealed distinct patterns. We found that in non-diabetic mice, females exhibited significantly higher basal EZH2 expression and H3K27me3 levels in proximal tubules compared with males. In diabetic mice, tubular EZH2 protein expression was significantly reduced in females, while no difference in *db/db* males was observed compared with the corresponding non-diabetic controls. However, the repressive mark H3K27me3 was reduced in diabetic mice irrespective of the sex, without any significant difference between *db/db* female and male mice. At present, the underlying mechanism responsible for the strong de-repressive effect of H3K27me3 observed in female *db/db* mice is not clear. One possible explanation is a higher expression of the H3K27me3 demethylase UTX in female *db/db* mice, which may contribute to reduced H3K27me3 levels independently of, or in addition to, reduced EZH2 expression. Of note, sex-specific differences of UTX expression have already been reported in mouse brain and neurons [72,73]. Moreover, as the UTX gene is located on the X chromosome and belongs to the genes that escape the X-inactivation [73,74], it may contribute to differences between males and females, which needs further investigation.

Therefore, our findings indicate that diabetic conditions are associated with a diminished expression of the repressive histone 3 mark H3K27me3 in proximal tubules, which may contribute to increased transcription of genes involved in the pathogenesis of DKD. The observed sex-related differences also highlight the fact that the influence of sex on the epigenetic modifications in diabetes should be studied not only in renal tissue but also in all organs and tissues that are affected from diabetes-related complications.

In summary, our data demonstrate that AGEs can regulate EZH2 and H3K27me3 in proximal tubular cells, leading to up-regulation of the mRNA expression of pro-fibrotic and pro-hypertrophic genes. Pharmacological inhibition of EZH2 by DZNep application reinforces these effects, suggesting a central role of EZH2/H3K27me3 in maintaining proximal tubular cell homeostasis. Furthermore, sex-specific differences in basal EZH2 and H3K27me3 levels suggest that female mice may be more resilient under non-diabetic conditions but more susceptible to epigenetic dysregulation during diabetes, which requires further investigation.

Our findings expand the understanding of the mechanisms linking AGEs to renal fibrosis, EMT, and tubular hypertrophy in diabetic nephropathy. Our study also highlights the importance of considering sex as a biological variable in studies of epigenetic regulation in the kidney. Targeting EZH2 and the H3K27me3 pathway may represent a therapeutic strategy to mitigate AGE-induced renal injury and progression of diabetic kidney disease.

## Data Availability

The data presented in this study are available on request from the corresponding author.

## Supplementary Materials

The following supporting information can be downloaded at https://www.mdpi.com/article/10.3390/cells14211729/s1: Table S1: Primer sequences used for semi-quantitative PCR or qRT-PCR amplification analyses. The Primer R-Squared (R^2^) and Efficiency (%) are shown for each primers pair. Table S2: Primary antibodies used for immunofluorescent stain or Western blot detection analyses. Table S3: Secondary antibodies used for immunofluorescent stain or Western blot detection analyses. Supplementary Figure S1: Protein expression of H3K27me3 in proximal tubule of non-diabetic and diabetic male mice. Representative images of all channels are shown. Scale bar: 50 µM. Supplementary Figure S2: Protein expression of H3K27me3 in proximal tubule of non-diabetic and diabetic female mice. Representative images of all channels are shown. Scale bar: 50 µM. Supplementary Methods: Script for analysis of the nuclei counting.
