# Supplementary material for: Advanced Glycation End Products Mediate Epigenetic Alteration of H3K27me3 in Renal Proximal Tubular Cells: Potential Role in Metabolic Memory
Source: Cells. 2025 Nov 4;14(21):1729. doi: 10.3390/cells14211729 (PMC12607550; doi:10.3390/cells14211729)
Supplement: Supplementary file 1 [file cells-14-01729-s001.zip › cells-3935467-Supplementary Method Script for cell counting.pdf]

Supplementary Method: Script for cell counting

As an example, here is the script for for counting the nuclei ifor EZH2 (channel=AF594). For H3K27me3 and NIPP1, only the names of the AF and the threshold were changed.
As the staining intensity was diffent for EZH2, H3K27me3 and NIPP1 stains an individual treshold was set up for each quantification.

Thresholds:

- EZH2: 7000
- H3K27me3: 4000
- NIPP1: 9000

```
import qupath.lib.common.GeneralTools
import qupath.lib.projects.Projects

def thresholdAF594 = 7000.0

def proj = getProject()
if (proj == null) {
   print "Run script with 'Run for Project'.\n"
   return
}
def baseDir = Projects.getBaseDirectory(proj)
def outCsv  = new File(baseDir, "af594_counts.csv")
if (!outCsv.exists()) {
   outCsv.text = "image,total_cells,af594_positive,measurement_key\n"
}

def imageData = getCurrentImageData()
if (imageData == null) {
   print "No image loaded.\n"
   return
}
def server  = imageData.getServer()
def imgName = GeneralTools.stripExtension(server.getMetadata().getName())
print "\nRunning: ${imgName}"

def example = getCellObjects().find()
if (example == null) {
   print "No cells detected.\n"
   outCsv << "${imgName},0,0,\n"
   return
}

def mList = example.getMeasurementList()
def names = mList.getMeasurementNames()
```

```
String keyAF594 = names.find { it == "Nucleus: AF594 max" }
if (keyAF594 == null) {
   keyAF594 = names.find { n ->
      n != null && n.toLowerCase().contains("af594") && n.toLowerCase().contains("max")
   }
}
if (keyAF594 == null) {
   keyAF594 = names.find { n ->
      if (n == null) return false
      def s = n.toLowerCase()
      (s.contains("af 594") || (s.contains("alexa") && s.contains("594"))) && s.contains("max")
   }
}

if (keyAF594 == null) {
   print "No measurement found for AF594 (Nucleus max).\n"
   outCsv << "${imgName},0,0,\n"
   return
}


int total = 0
int pos   = 0

for (cell in getCellObjects()) {
   def mm = cell.getMeasurementList()
   if (mm == null || !mm.containsKey(keyAF594)) continue

   def vObj = mm.get(keyAF594)
   if (vObj == null) continue
   double v = (vObj as Number).doubleValue()

   total++
   if (v > thresholdAF594) pos++
}

print "Total cells: " + total
print "AF594+ (Nucleus max > ${thresholdAF594 as int}): " + pos
print "Measurement key: " + keyAF594


outCsv << "${imgName},${total},${pos},\"${keyAF594}\"\n"

print "\n→ CSV updated: ${outCsv.getAbsolutePath()}\n"
```
