# Supplementary material for: Advanced Glycation End Products Mediate Epigenetic Alteration of H3K27me3 in Renal Proximal Tubular Cells: Potential Role in Metabolic Memory
Source: Cells. 2025 Nov 4;14(21):1729. doi: 10.3390/cells14211729 (PMC12607550; doi:10.3390/cells14211729)
Supplement: Supplementary file 1 [file cells-14-01729-s001.zip › cells-3935467-supplementary Table S1.pdf]

Supplementary Table S1. Primer's sequences used for semi-quantitative PCR for qRT-PCR amplification analyses. The Primer R-Squared (R^2) and Efficiency (%) are shown for each primers pair.

**Primers**	**Sequence (5'- 3')**	**Primer R-Squared** / **Primer Efficiency (%)**
Ctgf forward	CCTGGTCCAGACCACAGAGT	R-Squared 0.99;
Ctgf reverse	TTTTCCTCCAGGTCAGCTTC	Efficiency (%) 95.3
Ezh2 forward	CTGGAGGGAGCTAAGGAGT	R-Squared 0.99;
Ezh2 reverse	GTCCCTGCTTCTCTGTCACT	Efficiency (%) 100.8
Hprt forward	TGCTCGAGATGTCATGAAGG	R-Squared 0.99;
Hprt reverse	TATGTCCCCCGTTGACTGAT	Efficiency (%) 98.2
Nipp1 forward	CAGCACACATGGCACTTTCT	R-Squared 0.99;
Nipp1 reverse	TCCGCCCATCTTCTCATCTC	Efficiency (%) 102.8
p27 ^{Kip1} forward	CAG AAT CAT AAG CCC CTG GA	R-Squared 0.99;
p27 ^{Kip1} reverse	TCTGACGAGTCAGGCATTTG	Efficiency (%) 93.3
Rage forward	GCCGGTTTCTGTGACCCTGA	R-Squared 0.99;
Rage reverse	AGCCTGAAGGTGGAATAGTCG	Efficiency (%) 93.3
Snai1 forward	GCGGAAGATCTTCAACTGCAAATATTGTAA	R-Squared 0.96;
Snai1 reverse	GCAGTGGGAGCAGGAGAATGGCTTCTCAC	Efficiency (%) 105.12
Kdm6a forward	TGCTCCTCCATTACCATCCG	R-Squared 0.99;
Kdm6a reverse	GCTTTACGAGAGTCCTGGCA	Efficiency (%) 100.8
Kdm6a forward	TGCTCCTCCATTACCATCCG	R-Squared 0.99;
Kdm6a reverse	GCTTTACGAGAGTCCTGGCA	Efficiency (%) 100.8
