# Supplementary material for: Advanced Glycation End Products Mediate Epigenetic Alteration of H3K27me3 in Renal Proximal Tubular Cells: Potential Role in Metabolic Memory
Source: Cells. 2025 Nov 4;14(21):1729. doi: 10.3390/cells14211729 (PMC12607550; doi:10.3390/cells14211729)
Supplement: Supplementary file 1 [file cells-14-01729-s001.zip › cells-3935467-supplementary Table S2.pdf]

Supplementary Table S2. Primary antibodies used for immunofluorescent stain or Western blot detection analyses.

Antibody	Host species	Manufacturer	Dilution
EZH2	Rabbit, polyclonal	Invitrogen, Darmstadt, Germany 36-6300	IF 1:100
EZH2	Rabbit, polyclonal	Active Motif, Regensburg, Germany 39933	WB 1: 1000
GGT1	Rabbit, polyclonal	Invirtogen, Darmstadt, Germany, PA5-81395	IF 1:1000
H3K27me3	Rabbit, polyclonal	Merck KGaA, Darmstadt, Germany, CS-200603	WB 1:3000
H3K27me3 conjugated to Alexa Fluor® 647	Rabbit, monoclonal	Abcam, Cambridge, United Kingdom, ab270163	IF 1:100
Histone 3	Goat, polyclonal	Santa Cruz, Heidelberg, Germany, sc-8654	WB 1:500
NIPP1	Goat, polyclonal	Aviva Systems Biology, San Diego, CA, USA, OAEB01691	WB 1:1000
NIPP1	Rabbit, monoclonal	Aviva Systems Biology, San Diego, CA, USA, ARP40412_T100	IF 1:100
TATA-box-binding protein	Mouse, monoclonal	NSJ Bioreagents, San Diego, California, USA F52225	WB 1:1000
Vinculin	Mouse, monoclonal	Merck KGaA, Darmstadt, Germany, V9264	WB 1:1000
N-epsilon-(Carboxymethyl) Lysine (CML)	Rabbit, polyclonal	Cell Biolabs, Inc., CA , USA, STA-014	WB 1:1000
