# Supplementary material for: Advanced Glycation End Products Mediate Epigenetic Alteration of H3K27me3 in Renal Proximal Tubular Cells: Potential Role in Metabolic Memory
Source: Cells. 2025 Nov 4;14(21):1729. doi: 10.3390/cells14211729 (PMC12607550; doi:10.3390/cells14211729)
Supplement: Supplementary file 1 [file cells-14-01729-s001.zip › cells-3935467-supplementary Table S3.pdf]

Supplementary Table S3. Secondary antibodies used for immunofluorescent stain or Westeren blot detection analyses.

**Antibody**	**Host species**	**Manufacturer**	**Dilution**
Anti-rabbit IgG DyLight® 488	Goat	Vector Labs, California, USA	IF 1:500
Anti-rabbit IgG DyLight® 594	Goat	Vector Labs, California, USA	IF 1:1000
Anti-Rabbit IgG (H+L), Peroxidase-Labeled	Goat	SeraCare, Massachusetts, USA	WB 1:1000
Anti-Mouse IgG (H+L), Peroxidase-Labeled	Goat	SeraCare, Massachusetts, USA	WB 1:1000
Anti-Goat IgG (H+L), Peroxidase-Labeled	Rabbit	SeraCare, Massachusetts, USA	WB 1:1000
