# Supplementary figures and images for: Advanced Glycation End Products Mediate Epigenetic Alteration of H3K27me3 in Renal Proximal Tubular Cells: Potential Role in Metabolic Memory
Source: Cells. 2025 Nov 4;14(21):1729. doi: 10.3390/cells14211729 (PMC12607550; doi:10.3390/cells14211729)

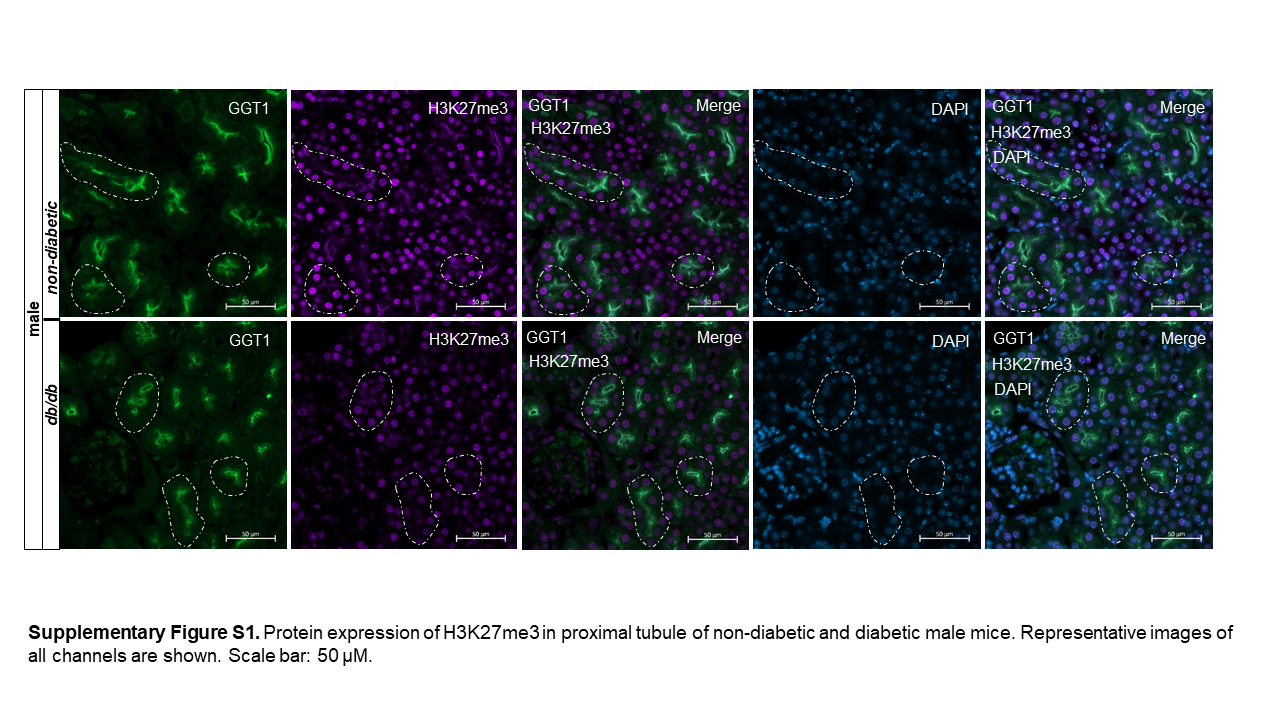

Supplement: Supplementary file 1 [file cells-14-01729-s001.zip › cells-3935467-supplementary Figure S1.tif]

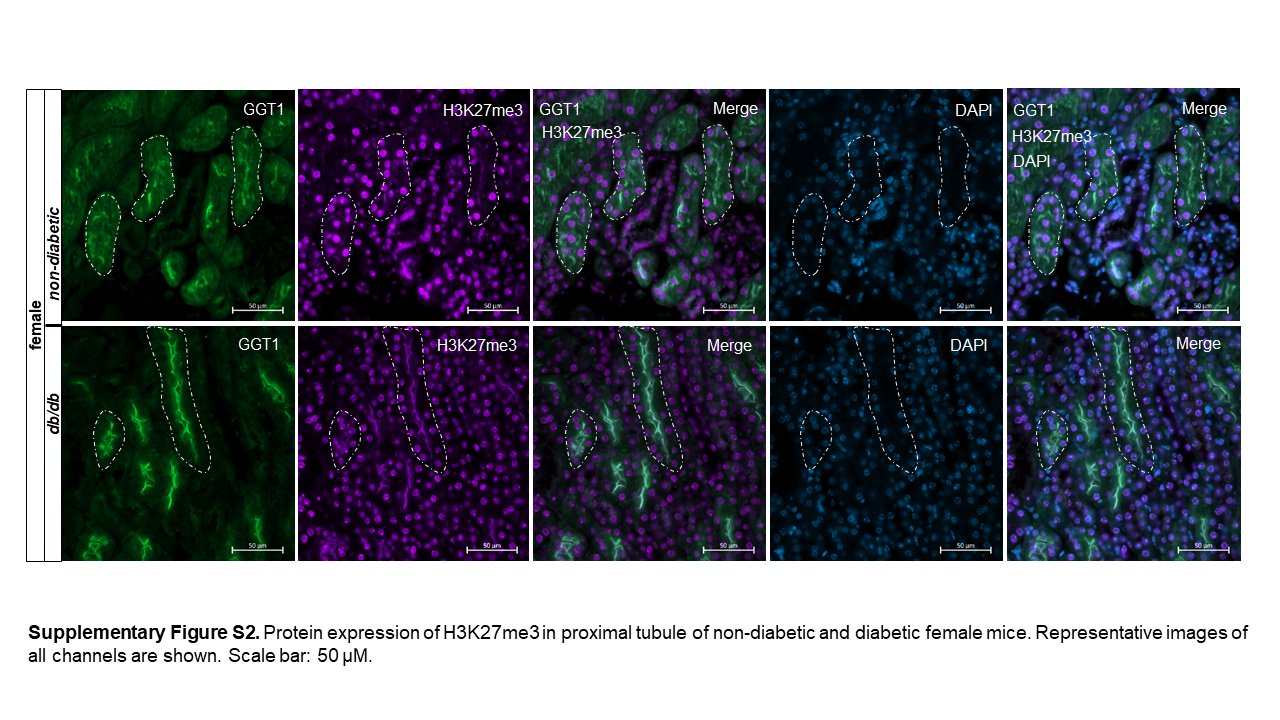

Supplement: Supplementary file 1 [file cells-14-01729-s001.zip › cells-3935467-supplementary Figure S2.tif]
